# Random Plant Viral Variants Attain Temporal Advantages During Systemic Infections and in Turn Resist other Variants of the Same Virus

**DOI:** 10.1038/srep15346

**Published:** 2015-10-20

**Authors:** Xiao-Feng Zhang, Jiangbo Guo, Xiuchun Zhang, Tea Meulia, Pierce Paul, Laurence V. Madden, Dawei Li, Feng Qu

**Affiliations:** 1 Department of Plant Pathology , Ohio Agricultural Research and Development Center, The Ohio State University, 024 Selby Hall, 1680 Madison Ave, Wooster, 44691, US; 2 State Key Laboratory of Agro-Biotechnology, College of Biological Sciences, China Agricultural University, Beijing, 100193, China; 3 School of Mathematics, Physics and Biological Engineering, Inner Mongolia University of Science and Technology, 27 Shaoxian Rd, Qingshan, Baotou, China; 4 Key Laboratory of Biology and Genetic Resources of Tropical Crops, ITBB, CATAS, Haikou, 571101, PR, China; 5 Molecular and Cellular Imaging Center, Ohio Agricultural Research and Development Center, The Ohio State University, 025 Selby Hall, 1680 Madison Ave, Wooster, 44691, US.

## Abstract

Infection of plants with viruses containing multiple variants frequently leads to dominance by a few random variants in the systemically infected leaves (SLs), for which a plausible explanation is lacking. We show here that SL dominance by a given viral variant is adequately explained by its fortuitous lead in systemic spread, coupled with its resistance to superinfection by other variants. We analyzed the fate of a multi-variant turnip crinkle virus (TCV) population in *Arabidopsis* and *N. benthamiana* plants. Both wild-type and RNA silencing-defective plants displayed a similar pattern of random dominance by a few variant genotypes, thus discounting a prominent role for RNA silencing. When introduced to plants sequentially as two subpopulations, a twelve-hour head-start was sufficient for the first set to dominate. Finally, SLs of TCV-infected plants became highly resistant to secondary invasions of another TCV variant. We propose that random distribution of variant foci on inoculated leaves allows different variants to lead systemic movement in different plants. The leading variants then colonize large areas of SLs, and resist the superinfection of lagging variants in the same areas. In conclusion, superinfection resistance is the primary driver of random enrichment of viral variants in systemically infected plants.

Understanding virus population dynamics in individual infected plants has far-reaching implications in management of virus diseases of crops. For instance, the level of genetic diversity of a virus is expected to correlate with its ability to jump into new hosts via viral variants retained in virus populations^1^,^2^. It is well understood that viruses with RNA genomes replicate through an error-prone process, and are hence thought to exist in host cells as populations consisting of large numbers of variants. On the other hand, earlier studies found that plants co-infected with multiple variants of the same virus often ended up containing substantially fewer variants than the inoculum^3^,^4^,^5^. Importantly, reduction in number of variants cannot be simply attributed to positive selection, as different sets of variants were recovered from different plants, or sometimes even in different tillers, branches, and leaf sections of the same plant^3^,^5^,^6^,^7^.

Exactly how plant hosts or viruses constrain the number of viral variants is not well understood. While some reports invoked plant antiviral defenses as possible driving forces, others suggested that certain virus-encoded functions might discourage secondary invasion of viral variants highly homologous to the ones already present in plants, thus could limit viral population sizes^3^,^8^,^9^. It was also suggested that cross-protection might play an important role in shaping population structures of RNA plant viruses^3^. Cross-protection refers to the specific protection against a virus in plants pre-inoculated with a mild isolate of the same virus^10^,^11^. Mechanistically, cross-protection was once thought to be caused by homology-based RNA silencing, although this notion has been challenged by several more recent studies^9^,^12^,^13^,^14^,^15^. RNA silencing-based defense enlists a complex set of proteins to combat intracellular parasites including viruses, retrotransposons, and other highly repetitive genome elements^16^. It is commonly triggered by intracellular occurrence of double-stranded RNA (dsRNA) or partially double stranded stem-loop RNA, which are processed by Dicer-like (DCL) nucleases into small RNAs of discrete sizes (21–25 nucleotides [nt]) referred to as small interfering RNAs (siRNAs). siRNAs then serve as sequence-specificity determinants of RNA-induced silencing complexes (RISCs), directing Argonaute (AGO) proteins to complementary RNA or DNA, silencing corresponding genes or genetic elements^17^,^18^.

Cross-protection may also be mechanistically related to superinfection resistance, also known as superinfection exclusion^9^,^19^. Superinfection resistance describes the inability of a virus to invade cells/tissues/organisms pre-infected by the same or a closely related virus, regardless of the severity of symptoms of the pre-existing virus^9^,^19^,^20^. Superinfection resistance was observed in both plant and animal virus infections, including several important human pathogenic viruses^21^,^22^,^23^. Studies with animal virus models suggested that resistance could occur at different steps of virus infection, including blockade of virus entry, or post-translational repression^21^,^22^. However, a possible relationship between superinfection resistance and the enrichment of a random few viral variants was not examined in these studies.

In the current report, we undertook a systematic investigation to uncover the mechanism of stochastic enrichment of a few viral variants during plant virus infections, and to evaluate its potential relationship with RNA silencing and superinfection resistance. To this end, we adopted as a new model the turnip crinkle virus (TCV), a small icosahedral virus with a single-stranded, nonsegmented RNA genome^24^. The positive sense TCV genome of 4,054 nt encodes five proteins, with the 5′ proximal P28 and its readthrough product (P88) implicated in viral genome replication. They are followed immediately by two small proteins (P8 and P9) essential for viral cell-to-cell movement, and the 3′ proximal P38 which is both the capsid protein (CP) and the viral suppressor of RNA silencing (VSR)^25^. Earlier studies established that TCV-targeting RNA silencing in *Arabidopsis* is initiated by the hierarchical actions of DCL4 and DCL2, and it is strongly suppressed by TCV-encoded VSR^24^,^25^,^26^,^27^,^28^. By contrast, DCL1 and DCL3, the two other *Arabidopsis* DCLs, played negligible (DCL3) or even antagonistic (DCL1) roles in anti-TCV RNA silencing^24^. Consequently, mutant *Arabidopsis* plants with both *DCL2* and *DCL4* knocked out (referred to as *dcl2 dcl4* plants) lack the ability to counteract TCV infections through RNA silencing^24^,^26^,^28^.

In order to unravel the underlying mechanism for stochastic enrichment of a few viral variants in infected plants, we followed the fate of an artificial TCV population containing nine distinct variants in 10 wild-type (wt), and 20 *dcl2 dcl4* mutant *Arabidopsis* plants. Our results suggest that the dominant variants in the systemic leaves (SLs) of a plant are likely those that reached these leaves the earliest. Once inside SLs, dominance of early arrivers is exacerbated by their ability to repress the replication of late arrivers in the same leaf areas through superinfection resistance. In summary, temporal variance in systemic colonization coupled with superinfection resistance adequately explains the stochastic dominance of a few variants in SLs.

## Results

### TCV variants constructed for the current study are similarly competent when introduced into plants separately

**Figure 1:**
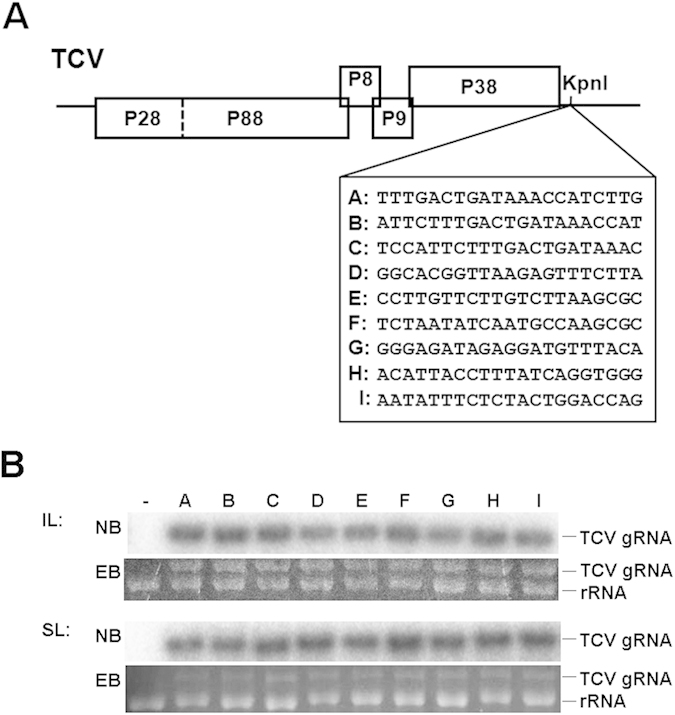
TCV genome, variants created in this study, and their infectivity in *Arabidopsis* plants. (**A**) Schematic representation of TCV genome organization, with the newly introduced KpnI site shown immediately downstream of the CP coding region. The variants A–I, each containing a 21 nt insert at the KpnI site, were depicted beneath the genome. (**B**) The accumulation levels of variant A–I in the inoculated and systemically infected leaves (ILs and SLs) of *Col-0* plants as determined by Northern blot hybridization (NB). The probe used was a 21-nt antisense oligo complementary to the CP coding region (sequence available upon request). EB, ethidium bromide-stained gels serving as loading controls.

To determine whether variants of TCV are stochastically excluded from SLs of infected plants, we first constructed a TCV population consisting of nine variants (A to I) by introducing a KpnI site immediately after the CP stop codon, and inserting nine different 21-nt fragments into this site (Fig. 1A). To ensure TCV infectivity was not compromised by these short inserts, variants were first brought into *Arabidopsis* plants separately, and their accumulation levels assessed. As shown in Fig. 1B, in both inoculated leaves (ILs) and SLs, genomic RNA of all nine variants accumulated to high levels typical of TCV infections, enabling their visualization in ethidium bromide (EB) stained gels, as well as easy detection with Northern blot (NB) hybridization using a TCV-specific probe (Fig. 1B, NB panels). Therefore, none of the nine variants was detectably handicapped in terms of local as well as systemic infectivity.

### Infections initiated with a mixture of nine variants lead to stochastic enrichment of a few variants in both wt and *dcl2 dcl4 Arabidopsis* plants

**Figure 2:**
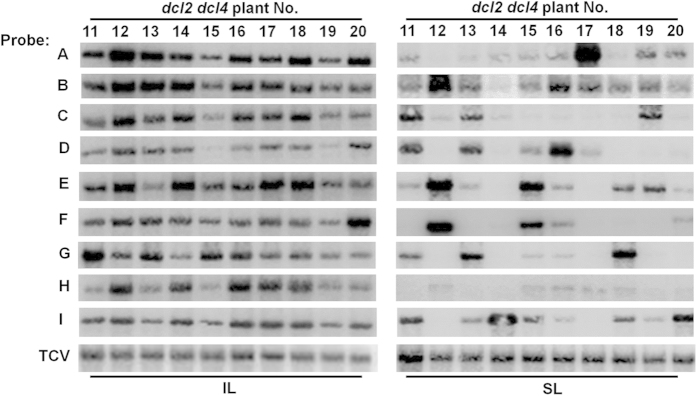
Stochastic enrichment of a few variants in 10 different *dcl2 dcl4* plants inoculated with a mixed inoculum containing an equal amount of nine different variants (A–I). On the left side, total RNA was extracted from ILs of the inoculated plants at 7 dpi and subjected to Northern blot hybridizations with probes indicated to the far left. On the right side, total RNA was extracted from SLs of the inoculated plants at 18 dpi and subjected to Northern blot hybridizations with probes indicated to the far left.

We next assessed how these nine variants would behave when introduced into plants as a mixed inoculum, and whether their behavior was influenced by RNA silencing-mediated antiviral defense. A mixed TCV inoculum containing an equal amount of infectious transcripts of the nine variants was mechanically inoculated to 10 wt and 10 *dcl2 dcl4* plants. Presence of each of the variants in ILs of all plants was confirmed with Northern blot hybridizations using nine different radioactively labelled oligonucleotides, each complementary to one of the nine inserts (data not shown. Also see Fig. 2, IL panels). The fate of the variants in SLs was then evaluated by subjecting the SLs of each of the infected plants to total RNA extractions at 18 dpi, followed by RT-PCR amplification of a TCV cDNA fragment encompassing the variant-specific region, and subsequent cloning of this fragment into a plasmid vector. The resultant recombinants were sequenced individually to reveal variant genotypes.

We first assessed the presence of different variants in SLs of individual plants, as well as their relative abundance, by sequencing 27 random clones per plant, with the results summarized in Tables 1 and 2. The per-variant counts were then subjected to statistical evaluation using a binomial probability test (http://stattrek.com/online-calculator/binomial.aspx), with 0.111 (1/9) as the expected probability, assuming equal competence of the nine variants. We recognize that relative competence of the variants in mixed infections might vary slightly, causing the expected probability to deviate from 0.111. However, these modest deviations were inconsequential as all variants except E had chance to dominate in SLs of at least one of the 20 plants (Tables 1 and 2). Furthermore, variant E could also become dominant in SLs of some plants upon inspection of more plants (see below and Fig. 2), thus illustrating a complete stochasticity of variants dominating SLs. The binomial probability test indicated that the numbers highlighted in bold in Tables 1 and 2 were significantly higher than expected (*p* < 0.01), confirming the dominance of underlying variants.

Two general trends emerged from the data in Tables 1 and 2. First, in almost all plants, there is a statistically significant enrichment of just one or two variant genotypes. Second, the enriched genotypes differed from plant to plant in a stochastic manner. In fact, nearly all variants had a chance to dominate the total counts in at least one plant (and variant E dominated *dcl2 dcl4* plants #12 and #15 as determined by Northern blot hybridizations shown in Fig. 2). Therefore, factors other than relative competence of the variants must have played a primary role in causing this stochasticity. Finally, these same trends persisted in *dcl2 dcl4* plants that lack anti-TCV RNA silencing activities (compare Tables 1 and 2), indicating that the role of RNA silencing in the stochastic enrichment of a few variants is minimal^24^,^26^.

### Stochastically enriched TCV variants co-exist with other less abundant variants in SLs

While one to two random variants dominated most of the examined SLs, up to six other variants were also detected at lower counts in all plants (e.g. plant #5 in Table 1 and plant #7 in Table 2). This raised the possibility that more variants could be detected by using more sensitive methods. We hence infected 10 additional *dcl2 dcl4* plants with the mixed inoculum and subjected the RNA samples isolated from ILs and SLs of individual plants to Northern blot hybridizations with variant-specific probes. All variants were detected in all ILs, although their levels varied (Fig. 2, IL panels). Note that the relative abundance of different variants in the same plant cannot be *directly* compared on Northern blots, due to variations in probe-labeling efficiencies and hybridization conditions. Nevertheless, the levels of most variants co-varied with each other in different IL blots, suggesting low levels of inter-variant competition within ILs. Importantly, blots of SLs confirmed the pattern of preferential enrichment of a few random variants in individual plants, as exemplified by the over-representation of variant A and underrepresentation of other variants in plant #17 (Fig. 2, SL panels, plant 17). Furthermore, by comparing the IL and SL blots, it is also evident that enrichment of a given variant in SL did not correlate with its relative level in IL. For example, variant A accumulated to comparable levels in ILs of the ten plants, yet it accumulated to drastically higher levels in the SL of plant #17 (Fig. 2, right panels). A similar case could be made for variants D, E, F, G, I in plants #16, #15, #12, #18, and #14, respectively. Therefore, factors other than variant abundance in ILs played a more prominent role in the enrichment of a random few variants in SLs.

A more crucial revelation is that notwithstanding of enrichment of a few, most other variants were present in SLs of most plants at lower levels. For example, all nine variants were detected in plants #15 and 16, and eight out of nine were detected in plants #13 and 20 (Fig. 2, SL panels). In fact, the fewest number of variants detected in a given plant was six out of nine, in plant #12. Therefore, while only a few of the variants dominated SLs in a stochastic manner, most of the other co-introduced variants could enter and multiply in SLs to certain extents. As we will show below, this could reflect the relatively small differences in the timing of systemic spread among co-introduced variants.

### Sequential introduction of different variant mixes allows earlier variants to exclude the later ones from SLs

If all variants introduced through a mixed inoculum could access SLs, what could have caused the preferential enrichment of a few variants? Could the timing of SL entry be one of the factors? To test this possibility, we created a subpopulation by mixing variants F, G, H, and I (FGHI), and paired the FGHI subpopulation with variant A in a series of sequential inoculations. Fewer variants were used in this set of experiments to simplify the subsequent analyses with Northern blot hybridizations. For sequential inoculations, ILs of the same *dcl2 dcl4* leaves were divided into two halves (proximal vs. distal) with a Sharpie pen, and immediately inoculated on the proximal halves with one of the variant sets (variant A or FGHI mix). After a 48 hour delay, the second inoculum was applied on the distal halves of ILs. The 48-hour interval was initially chosen because previous studies showed that most viruses needed two days of cell-to-cell movement before transiting to systemic movement^29^.

**Figure 3:**
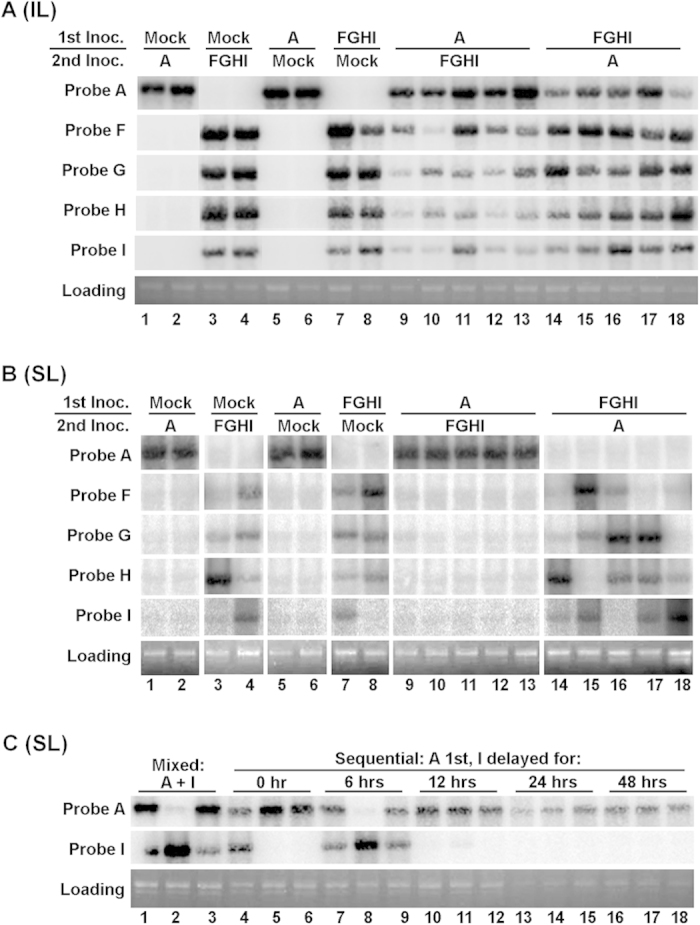
Repression of TCV variants by their pre-inoculated counterparts. (**A,B**) A, F, G, H, and I are TCV variants differing at a 21 nt region (Fig. 1A). Either variant A or an FGHI variant mix was used to inoculate the proximal half of an IL, followed by a secondary inoculation 48 hours later with the reciprocal variant sets on the distal half of the same IL. Total RNA samples were then collected from ILs (**A**) and SLs (**B**) of five independent plants inoculated with A/FGHI (lane 9–13) and FGHI/A (lanes 14–18), and two independent plants of each of the control groups. These samples were then separated on a denaturing agarose gel, transferred to Nylon membranes, and subjected to hybridizations with 32P-labeled oligo probes specific for each variant. (**C**) Sequential inoculations were repeated with variants A and I, with I delayed for o, 6, 12, 24, and 48 hours, respectively.

We first examined ILs to ensure both inocula led to successful infections. As shown in Fig. 3A, although the accumulation levels of secondary variants appear to be modestly reduced when compared with the same variants introduced as the primary inocula (compare lanes 9–13 with 14–18 for variant A, and vice versa for variants F, G, H, and I), all variants were clearly detectable in ILs. However, in SLs, prior inoculation with variant A completely abolished the accumulation of all four secondary variants (Fig. 3B, lanes 9–13 of rows 2–5. Note the absence of F, G, H, or I signals in these lanes). Conversely, prior inoculation with the FGHI mix blocked the accumulation of variant A (Fig. 3B, lanes 14–18 of row 1). Prior mock inoculation did not prevent the accumulation of secondary variants (Fig. 3B, lanes 1–4). Together these results demonstrated that pre-introduced TCV variants exerted a robust repression on secondary TCV variants through a mechanism independent of RNA silencing.

We next attempted to determine the shortest temporal delay needed to ensure a complete dominance of the first variant. To do this, we repeated the sequential inoculations with variants A and I in five groups *dcl2 dcl4* plants, with the secondary inoculation delayed for 0, 6, 12, 24, and 48 hours, respectively (Fig. 3C). An (A + I) mixed infection was also included as a control (Fig. 3C, lanes 1–3). The reason for using just two variants (A and I) in this experiment is that, as shown in Fig. 3B, a single precedent variant (A) was fully capable of excluding multiple other variants, and it could also be completely excluded by other early arrivers (Fig. 3B, top row, lanes 9–18). Consistent with earlier results, (A + I) mixed inoculations led to dominance by I in one plant (Fig. 3C, lane 2), and A in two (lanes 1 and 3). Similarly stochastic dominance by either variant was also observed in plants in which the variant I was introduced at zero or six hours later than A (Fig. 3C, lanes 4–9). However, a 12-hour interval was enough to cause consistent dominance of the earlier variant (A) over the later one (I) (lanes 10–18). Note here that variant A was unlikely to be substantially more competent than variant I, as demonstrated by the complete exclusion of variant A by the pre-introduced FGHI mix (Fig. 3B). Together these results strongly suggest that dominance by a few TCV variants in SLs is likely due to their earlier SL colonization and active repression of their late arriving counterparts.

### TCV variants specifically repress other variants of the same virus

We next wondered whether dominance by TCV in SLs affected the fate of other co-infected virus species. To test that, we used the same sequential inoculation procedure to assess if a TCV variant could prevent the infection of carnation mottle virus (CarMV), a virus in the same genus as TCV, yet sharing limited sequence similarity [50–55% at the amino acid (aa) levels]. We conducted this experiment in *N. benthamiana* as CarMV does not infect *Arabidopsis*. Furthermore, since CarMV replicated to relatively low levels in *N. benthamiana*, we used *Nb*-P19, a transgenic *N. benthamiana* line expressing the P19 VSR of tomato bushy stunt virus, to minimize the differences in accumulation levels between TCV variants and CarMV.

**Figure 4:**
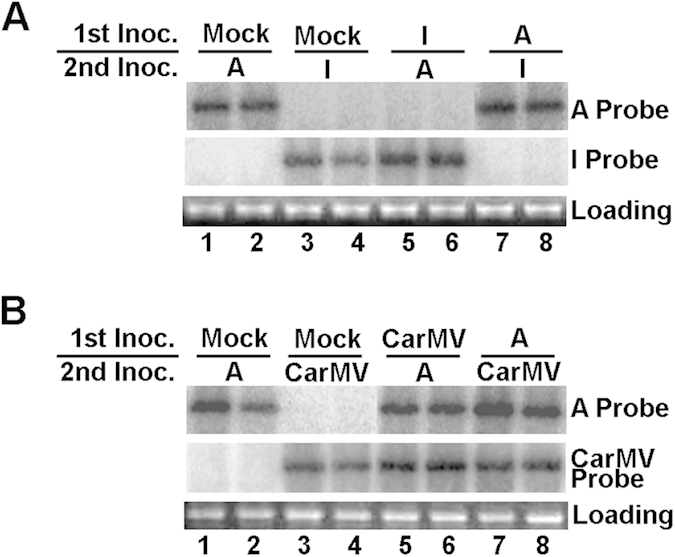
Exclusion of a TCV variant by its pre-inoculated relative is highly specific. (**A**) Mutual exclusion between sequentially inoculated variants A and I in *N. benthamiana* plants. (**B**) Lack of exclusion between TCV variant A and CarMV. Sequential inoculations were performed as described earlier, except here the *N. benthamiana* plants were used as hosts. Northern blot hybridizations were carried out to distinguish the various virus variants.

We first reproduced the mutual exclusion between TCV variants in *N. benthamiana* by sequentially inoculating variants A and I onto two halves of the same *Nb*-P19 leaves. As expected, prior inoculation with I (Fig. 4A, lanes 5 and 6) or A (lanes 7 and 8) prevented the accumulation of A or I, respectively, in the SLs of infected plants. However, similar experiments with variant A and CarMV revealed that they coexisted in the sequentially infected plants, regardless of the order of inoculation (lanes 5–8). Thus, exclusion between TCV variants likely depended on high levels of sequence identity at either nt or aa levels.

### Exclusion among TCV variants occurs at the sites where they meet each other

**Figure 5:**
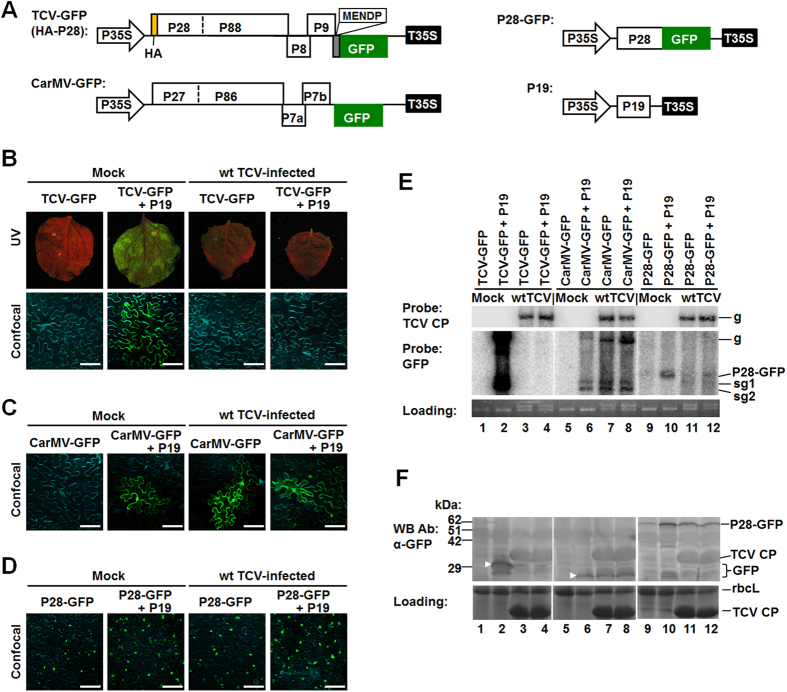
Exclusion of a secondary TCV variant occurs in the SLs. (**A**) Schematic representation of the constructs used in this set of experiments. TCV-GFP (HA-P28) was based on a previously reported construct in which the cycle 3 GFP coding sequence was fused to the first five amino acid residues of TCV CP. An HA epitope tag was additionally fused to the N-terminus of P28 to facilitate the detection of P28/P88 proteins. CarMV-GFP was generated from CarMV cDNA in which the N-terminal 2/3 of the CP coding sequence was replaced by that of sGFP through a PCR-based procedure. P28-GFP is a transient expression construct designed to express the P28 replication protein of TCV fused to the N-terminus of GFP. All constructs used in these experiments were under control of the 35S promoter and terminator (P35S and T35S), and mobilized into a pPZP212-based binary vector to facilitate agro-infiltration. (**B**) Macroscopic (under UV light) and microscopic (confocal microscopy) images of *N. benthamiana* leaves infiltrated with TCV-GFP or TCV-GFP + P19. The plants that were either healthy controls (left two panels) or pre-infected with wt TCV (right two panels). (**C**) Confocal images of *N. benthamiana* leaves infiltrated with CarMV-GFP or CarMV-GFP + P19. As in (**B)**, the plants were either healthy controls (left two panels) or pre-infected with wt TCV (right two panels). (**D**) Confocal images of *N. benthamiana* leaves infiltrated with P28-GFP or P28-GFP + P19. For (**B**–**D**) the cyan-colored cell boundaries arose from staining with DAPI that visualizes cell walls. The size bar = 100 μm. (**E**) The levels of wt TCV gRNA and that of TCV-GFP, CarMV-GFP, P28-GFP in the varying treatments revealed by Northern blot hybridizations with a TCV CP-specific and a GFP-specific probe. The loading control is an EB-stained gel showing both 25S rRNA and wt TCV gRNA where visible. (**F**) The protein levels of GFP (lanes 1–8) and P28-GFP (lanes 9–12) revealed by Western blot analysis with a GFP antibody. Note that the larger size of GFP produced by TCV-GFP is likely caused by the N-terminal 5-aa fusion, and/or the aa sequence differences between cycle 3 GFP (in TCV-GFP) and sGFP (in CarMV-GFP and P28-GFP). Also note that TCV CP produced by wt TCV was visible on the Coomassie blue-stained loading control, and on Western blots as a thick nonspecific band. The HA-tagged P28 and P88 produced by TCV-GFP are shown in an additional blot at the bottom.

Our results so far led us to hypothesize that the non-dominating variants did enter the leaves occupied by the dominating ones but were prevented from expanding themselves in the same leaf sections occupied by the former. To test this hypothesis directly, we first initiated systemic, wild-type (wt) TCV infections in *N. benthamiana* plants, and then delivered a GFP-tagged TCV variant [Fig. 5A, TCV-GFP (HA-P28), simplified as TCV-GFP hereafter] onto symptomatic SLs of these plants using *Agrobacterium*-mediated delivery (agro-infiltration). An *Agrobacterium* strain harboring a P19-expressing plasmid was co-delivered in some treatments to counteract RNA silencing as the TCV VSR (CP) was replaced by GFP in TCV-GFP. P19 was chosen over TCV CP to simplify the interpretation as the latter could itself be targeted by RNA silencing triggered by the pre-existing wt TCV. As shown in Fig. 5B, while in control *N. benthamiana* leaves TCV-GFP replication as evidenced by GFP fluorescence was easily detectable in the presence of the P19 VSR (Mock/TCV−GFP + P19), it was completely abolished in leaves with pre-existing wt TCV infections. To further assess the specificity of this interference, we followed the fate of a similarly engineered CarMV-GFP construct in the same type of SLs. As shown in Fig. 5C, CarMV-GFP replicated to similar levels on both healthy and TCV-infected leaves, as long as RNA silencing is suppressed by P19 or TCV CP provided through wt TCV pre-infection (Fig. 5C, 2^nd^ to 4^th^ panels), indicating that pre-existing TCV specifically stops the multiplication of another TCV variant, but not a more distant virus, on the same leaves.

To additionally assess whether the specific repression was caused by RNA silencing, we delivered a non-replicating construct designed to transiently express the GFP-tagged P28 protein of TCV (P28-GFP). Here P28 was chosen because its 750-nt region accounts for almost 20% of TCV genome, encompassing a number of highly accumulating TCV siRNAs^30^. The expression of P28-GFP was detected at similar levels in both mock and wt TCV-infected cells, as small, brightly green aggregates under a confocal microscope (Fig. 5D). The persistent P28-GFP expression in wt TCV-infected leaves suggests that wt TCV-triggered RNA silencing alone could not have blocked the replication of TCV-GFP.

These results were further verified by Northern blot hybridizations. As shown in Fig. 5E (top panel), wt TCV gRNA was consistently detected in the SLs of TCV-pre-infected plants (lanes 3, 4, 7, 8, 11, and 12). By contrast, TCV-GFP gRNA as well as sgRNAs were only detectable (with a GFP-specific probe) in leaves of mock plants co-infiltrated with TCV-GFP and P19 (Fig. 4E, middle panel, lane 2), but not in SLs of wt TCV-pre-infected plants (lanes 3 and 4). Importantly, this repression was not caused by simple competition between viruses, as CarMV-GFP replicated to easily detectable levels in the presence of P19, wt TCV, or both (lanes 5–8). Notably, when compared with mock leaves, the wt TCV-pre-infected SLs did cause a measurable reduction of P28-GFP mRNA levels (compare lanes 10 and 12), suggesting that P28-GFP mRNAs were partially susceptible to siRNAs derived from wt TCV infections. However, this did not abolish the accumulation of P28-GFP protein, as indicated by confocal microscopy (Fig. 5D), as well as Western blotting (Fig. 5F, lanes 9–12). Therefore, RNA silencing originated from wt TCV was unable to abolish the expression of P28-GFP. Consequently, the complete shut-down of TCV-GFP by pre-existing wt TCV was unlikely caused by RNA silencing.

Western blotting also detected GFP in (Mock/TCV−GFP + P19) samples, (Mock/CarMV-GFP + P19) samples, and (wt TCV/CarMV-GFP) samples (with or without P19) (Fig. 5F, lanes 2, 6–8, white arrows). The bigger size of GFP associated with TCV-GFP infections is likely due to the five extra N-terminal aa it inherited from TCV CP ORF (Fig. 5A), or the different GFP variants used (cycle 3 GFP^31^ in TCV-GFP versus sGFP^32^ in CarMV-GFP and P28-GFP). In conclusion, the highly specific repression of secondary TCV variants by their pre-existing counterparts occurred in the leaves they encounter each other, and it could not be adequately explained by RNA silencing.

## Discussion

Understanding how virus populations oscillate in host plants should not only advance our basic knowledge of virus evolution, but also enables improved management of crop virus diseases. Previous studies using a variety of systems suggest that the systemic movement stage serves as a population bottleneck to dramatically reduce the population size of RNA viruses but not DNA viruses^3^,^4^,^5^,^33^. However, exactly how this occurs remains to be resolved. In the current study, we used the TCV-*Arabidopsis* model system to investigate the dynamics of virus populations in individual host plants. A number of noteworthy observations emerged from our experiments. We found that in ILs of both wt and *dcl2 dcl4* plants, all variants were easily detectable regardless of the manner of introduction, suggesting relatively mild inter-genotype competition at the primary infection site. In contrast, all SLs experienced substantial enrichment of a few variants, with different variant genotype(s) enriched in different plants. Overall these findings agreed with previous observations made with other plant RNA viruses^3^,^4^,^5^, and reinforce the notion that RNA virus populations undergo uneven enrichment of random variants during or after systemic movement.

Notably, the same pattern of stochastic enrichment of a few variants persisted in mutant plants that lacked effective RNA silencing-mediated antiviral defense (*dcl2 dcl4* plants), thus ruling out a prominent role of RNA silencing in this process. This novel insight is significant because until recently RNA silencing was considered the primary mechanism that targets secondary infections by highly homologous viruses through cross-protection^12^,^13^. Consistent with our results, several studies by Ziebell and colleagues^14^,^15^ likewise refuted an active role of RNA silencing in cross-protection. In summary, if stochastic variant enrichment indeed shares the same mechanism(s) with at least some forms of cross-protection, as suggested by Hall and colleagues^3^, then the notion of RNA silencing as sole mechanism of cross-protection must be reconsidered.

Importantly, we were able to simulate the dominance of a few variants in SLs by inoculating two halves of the same IL with different variants, at different time points. Indeed, a mere 12 hour head-start led to a complete dominance by the first variant. This suggests that enrichment of a few variants in SLs of plants inoculated with a mixed virus population could have resulted from earlier arrival of these variants in SLs. Given the random distribution of primary infection foci on ILs, some of these foci can be expected to expand into vascular bundles earlier than others, or with larger virion numbers, causing the corresponding variants to dominate SLs. The stochastic nature of dominance in different plants could thus be explained as variants with a lead in systemic movement are expected to vary from plant to plant unpredictably. Most importantly, we show that SLs pre-infected with wt TCV robustly repressed the multiplication of a secondary TCV variant at the SL site of their encounter in a highly specific, yet largely RNA silencing-independent manner. This repression could in turn exacerbate the systemic movement advantage enjoyed by a few random variants.

**Figure 6:**
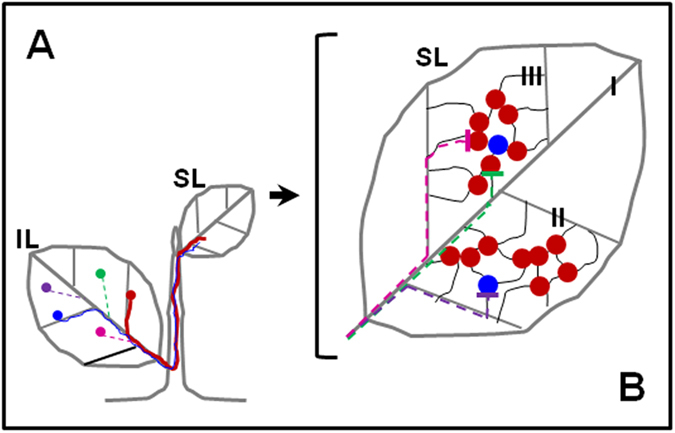
A model for the reduction of viral population size as the infection moves from an IL to an SL. (**A**) Infection foci formed by different viral variants are represented by colored dots on IL. The variant represented by the red dot expands right into a leaf vein and is expected to release a large amount of progenies for systemic movement (depicted by a thick solid red line extending to the SL) at the earliest time point. On the other hand, the blue variant is located slightly off a leaf vein and thus is expected to enter systemic movement phase in a smaller amount initially (depicted as a thin solid blue line). Other variants are expected to transit to the systemic movement phase at later time points (depicted as dashed lines). Note here that the use of major veins is purely for illustration purpose and does not imply that the entry of viruses into vascular bundles is exclusively through these veins. (**B**) In the SL, the larger amount of progenies of the red variant initiated more infection niches than the blue variant as they exit vascular bundles through the junctions of tertiary vein network (type III veins). Other variants, represented by dashed lines blocked at the sites of red and blue variants, are expected to be excluded from the niches already occupied by the early arrivers via as-yet-unknown mechanisms.

Together our data support a new model that accounts for both the enrichment of a few viral variants in SLs of any single plant, and the stochastic nature of dominant variants in different plants. As shown in Fig. 6, this model postulates that different viral variants establish independent, random infection foci on ILs (depicted as colored dots in Fig. 6A) that expand until they gain access to vascular bundles, where they transit to systemic movement. Due to differences in their easiness to access vascular bundles, but also in their relative competitiveness, some variants, depicted as a red dot in Fig. 6A, will transit to systemic movement sooner than others (thick red line in Fig. 6A), and then establish the first wave of systemic infection niches in SLs of the plant (multiple red dots in Fig. 6B). Likewise, a smaller amount of virions of a different variant, depicted as blue dots and lines in Fig. 6, could enter the same SL at about the same time to establish its infection niches in a smaller portion of the SL, provided that its primary infection focus is slightly more removed from vascular bundles, or the corresponding variant is slightly less robust. These earlier infection niches would then actively resist the reproduction of late arriving variants (green, purple, and pink dots and dotted lines in Fig. 6) through superinfection resistance. Assuming all variants in the mixed inoculum are similarly competitive, it is expected that the order by which different variants reach vascular bundles would vary from plant to plant in an entirely stochastic manner, accounting for plant-to-plant variations in the identities of dominant variants.

We wish to highlight an earlier study by Roberts and colleagues^34^ that established that, upon arriving at SLs, intact virions of potato virus X (PVX) exit vascular bundles of *N. benthamiana* plants almost exclusively at sites where two or more of tertiary (class III) veins converge (overlaid by red and blue dots in Fig. 6B). Similar exit preference for TCV in *Arabidopsis* plants has been documented by us^27^. Although the total number of class III vein junctions in a given leaf is expected to be high, it is nevertheless finite. Accordingly, the number of SL sites at which virions exit from vascular bundles is also limited. As a result, a sufficiently large number of early arriving variant could saturate these exit sites and colonize the adjacent leaf areas, and effectively resist the subsequent superinfection by their late-arriving cousins. Conversely, if the temporal lead of early arriving variants is relatively small, as in mixed infections, the late-arriving variants could still establish their smaller infection niches before the earlier ones occupy the entire leaf.

In addition to providing satisfactory explanations for findings of the current study, this model predicts that in leaves where co-existence among different variants do occur, the co-existing variants would form their own infection “islands” separate from each other thanks to superinfection resistance. In fact, this has been elegantly demonstrated by a number of earlier reports using modified viruses that express different fluorescent protein tags^35^,^36^,^37^. By extension, this model is also consistent with previous reports showing that different branches/tillers, leaves, or leaf sections contained different sets of variants^3^,^4^,^5^,^6^,^7^,^33^. Additionally, this model is in agreement with a study by Zwart and colleagues^8^ showing that at higher infection doses both of the co-introduced virus variants have similar chances to reach systemic leaves, as higher doses would increase the odds of both entering vascular bundles simultaneously. Finally, this model predicts that, if one viral variant is brought into plants ahead of other variants of the same virus, allowing it to establish precedent systemic infection, it would go on to dominate or even become the sole detectable variant in the SLs. Therefore, it is also consistent with the superinfection resistance phenomenon observed by Folimonova and colleagues^9^,^19^,^20^, and at least some forms of cross-protection.

## Methods

### Constructs

The pTCV (previously T1d1) construct has been described^25^. A KpnI site was created in pTCV after the CP stop codon, at nucleotide (nt) position #3803–3808, resulting in TCV-KpnI (Fig. 1A). Nine different 21 nt fragment were then inserted at the KpnI site to create variants A – I (Fig. 1A). All constructs were sequenced to confirm their identities. The TCV-GFP construct reported in earlier studies^25^,^27^ was modified in the current study by fusing an HA-epitope tag to the N-terminus of P28 through overlapping RT-PCR. The resulting TCV-GFP (HA-P28) construct replicated in *N. benthamiana* cells to levels indistinguishable from the original TCV-GFP (data not shown). The CarMV-GFP was produced by replacing the N-terminal 2/3 of the CarMV CP coding sequence with that of sGFP through overlapping PCR. The P28-GFP construct was similarly produced. TCV-GFP (HA-P28), CarMV-GFP, P28-GFP cDNAs were then sandwiched between P35S and T35S and mobilized into a pPZP212-based binary vector for use in agro-infiltrations using previously described procedures^25^. The P19 construct was from an earlier study^25^.

### Plant materials

The sources of Col-0 and *dcl2 dcl4* mutant *Arabidopsis* plants have been described previously^24^. Both *Arabidopsis* and *N. benthamiana* plants were reared in growth chambers or a growth room with the temperature set at 22 ^o^C. The day length was 14 hours.

### Infection of *Arabidopsis* and *N. benthamiana* plants with *in vitro* transcripts

*In vitro transcripts* of TCV variants (A–I), as well as that of CarMV, were produced using the TranscriptAid T7 High Yield Transcription Kit (Fermentas, Glen Burnie, MD), and purified according to the kit’s instruction. The integrity of the transcripts was examined with agarose gel electrophoresis. For mixed infections, an equal amount (10 μg) of transcript RNA was withdrawn from each purified transcript and combined with each other to make the mixed inoculum. The mixed inoculum was further diluted to 10 ng/μl with a inoculation buffer containing 50 mM glycine, 30 mM K2HPO4, pH 9.2, 1% bentonite, and 1% celite. For mechanical inoculation of *Arabidopsis* and *N. benthamiana* leaves, 20 μl of this 10 ng/μl inoculum was spotted on each leaf and gently spread with a gloved finger or a Q-tip.

### Agro-infiltration of *N. benthamiana* plants

Agro-infiltration was used to initiate the superinfection of TCV-GFP and CarMV-GFP on N. benthamiana leaves systemically infected with wild-type TCV. The details for agro-infiltration were given in ref 24.

### RNA blot analysis

Total RNAs were extracted from infected or infiltrated plants and subjected to RNA blot analysis to detect TCV viral RNAs, or GFP mRNA using published protocols^25^,^27^,^28^. Variant-specific probes were generated by end-labeling oligonucleotides complementary to the corresponding variant inserts with radioactive gamma ^32^P ATP and T4 polynucleotide kinase (Fermentas).

### Sequence analysis

From every TCV-infected *Arabidopsis* plant, two young rosette leaves were collected and subjected to RNA extraction separately. Typically we chose the youngest leaves among the ones that are one centimeter or greater in length. The total RNA samples were then subjected to RT-PCR with primers TCV-3312F (5′-CAGATTCTACTGACCGCTTTG-3′) and TCV-3997R (5′-ACAGCCCACCCTTTCGGGAT-3′) to amplify a 686 bp TCV cDNA fragment encompassing the 21 nt insertions. The PCR products were then cloned into pBlueScript SK. Individual clones were randomly selected and sent to Eurofins (Huntsville, AL) for sequencing.

### Statistics

The occurrence of a given viral variant clone among all clones sequenced was treated as binomial events, and the observed frequencies were used to calculate the binomial probability relative to an approximated expected frequency of 0.11 (1/9) (http://stattrek.com/online-calculator/binomial.aspx). The bold numbers in Tables 1 and 2 had a calculated p value smaller than 0.01, thus considered to be significantly higher than the expected counts.

### Western blot analysis

Protein extracts were prepared from agro-infiltrated or virus-infected plant tissues using a routine procedure^27^. The anti-GFP antibody was purchased from Sigma-Aldrich.

### Confocal microscopy

Confocal microscopic observations were carried out using a Leica Confocal microscope (TCS SP5) available through Molecular and Cellular Imaging Center at the Ohio Agricultural Research and Development Center, The Ohio State University^38^.

## Additional Information

**How to cite this article**: Zhang, X.-F. *et al.* Random Plant Viral Variants Attain Temporal Advantages During Systemic Infections and in Turn Resist other Variants of the Same Virus. *Sci. Rep.* **5**, 15346; doi: 10.1038/srep15346 (2015).

## Supplementary Material

Supplementary Information

## Figures and Tables

**Table 1:** Number of cDNA clones per variant derived from SL samples of Col-0 plants infected with a mixed TCV inoculum containing nine variants.

TCV variants	SL of individual *Col-0* plants									
	#1	#2	#3	#4	#5	#6	#7	#8	#9	#10
A	1				4	**8**	6	1	**18**	
B	1		**10**		1		1	**18**		**9**
C				4		**10**	1		2	
D			6		1					
E	1			2	1					6
F	**10**									
G	2	2	3		2	4	**17**			
H		3		**12**	**10**					
I	**12**	**22**	**8**	**8**	**8**	4		6	7	**12**
Total	27	27	27	26	27	26	25	25	27	27

TCV-specific cDNA encompassing the 21-nt variant-specific region was generated from SL samples collected from each of the ten infected plants, cloned in a plasmid vector. Subsequently, 27 clones were sequenced for every plant. The numbers in bold reflect statistically significant deviation from the expected detection rate (see Materials and Methods. The cut-off p value was 0.01).

**Table 2:** Number of cDNA clones per variant derived from SL samples of *dcl2 dcl4* plants infected with a mixed TCV inoculum containing nine variants.

TCVVariants	SL of individual *dcl2 dcl4* plants									
	#1	#2	#3	#4	#5	#6	#7	#8	#9	#10
A	**10**	2		**11**	6	2		**10**	**7**	
B				2	5	1			1	3
C			**17**	**8**		1	3	**11**	3	**13**
D	**16**		**10**	1	**10**	3	**13**			
E							1	1		5
F				1		**11**	2			
G						4	1			
H		4		2	5			1	3	1
I	1	**19**	1		2		5	2	**8**	
Total	27	25	28	25	28	22	25	25	22	22

TCV-specific cDNA encompassing the 21-nt variant-specific region was generated from SL samples collected from each of the ten infected plants, and cloned in a plasmid vector. Subsequently, 27 clones were sequenced for every plant. The numbers in bold reflect statistically significant deviation from the expected detection rate (see Materials and Methods. The cut-off p value was 0.01).

## References

[b1] Fargette D. *et al.* Molecular ecology and emergence of tropical plant viruses. Annual Review of Phytopathology 44, 235–260 (2006). 10.1146/annurev.phyto.44.120705.104644 16784403

[b2] Elena S. F. *et al.* The evolutionary genetics of emerging plant RNA viruses. Molecular Plant-Microbe Interaction 24, 287–293 (2011). 10.1094/MPMI-09-10-0214 21294624

[b3] Hall J. S., French R., Hein G. L., Morris T. J. & Stenger D. C. Three distinct mechanisms facilitate genetic isolation of sympatric *Wheat streak mosaic virus* lineages. Virology 282, 230–236 (2001). 11289805 10.1006/viro.2001.0841

[b4] Sacristán S., Malpica J. M., Fraile A. & García-Arenal F. Estimation of population bottlenecks during systemic movement of *Tobacco mosaic virus* in tobacco plants. Journal of Virology 77, 9906–9911 (2003). 12941900 10.1128/JVI.77.18.9906-9911.2003 PMC224588

[b5] Li H. & Roossinck M. J. Genetic bottlenecks reduce population variation in an experimental RNA virus population. Journal of Virology 78, 10582–10587 (2004). 15367625 10.1128/JVI.78.19.10582-10587.2004 PMC516416

[b6] French R. & Stenger D. C. Population structure within lineages of *Wheat streak mosaic virus* derived from a common founding event exhibits stochastic variation inconsistent with the deterministic quasi-species model. Virology 343, 179–189 (2005). 16181653 10.1016/j.virol.2005.08.037

[b7] Jridi C., Martin J.-F., Marie-Jeanne V., Labonne G. & Blanc S. Distinct viral populations differentiate and evolve independently in a single perennial host plant. Journal of Virology 80, 2349–2357 (2006). 16474141 10.1128/JVI.80.5.2349-2357.2006 PMC1395380

[b8] Zwart M. P., Daròs J.-A. & Elena S. F. One is enough: *in vivo* effective population size is dose-dependent for a plant RNA virus. PLoS Pathogens 7, e1002122 (2011). 21750676 10.1371/journal.ppat.1002122 PMC3131263

[b9] Folimonova S. Y. Superinfection exclusion is an active virus-controlled function that requires a specific viral protein. Journal of Virology 86, 5554–5561 (2012). 22398285 10.1128/JVI.00310-12 PMC3347309

[b10] Chewachong G. M. *et al.* Generation of an attenuated, cross-protective Pepino mosaic virus variant through alignment-guided mutagenesis of the viral capsid protein. Phytopathology 105, 126–134 (2015). 25496364 10.1094/PHYTO-01-14-0018-R

[b11] Ziebell H. & Carr J. P. Cross-protection: a century of mystery. Advances in Virus Research 76, 211–264 (2010). 20965075 10.1016/S0065-3527(10)76006-1

[b12] Ratcliff F., Harrison B. D. & Baulcombe D. C. A similarity between viral defense and gene silencing in plants. Science 276, 1558–1560 (1997). 18610513 10.1126/science.276.5318.1558

[b13] Baulcombe D. RNA silencing in plants. Nature 431, 356–363 (2005). 15372043 10.1038/nature02874

[b14] Ziebell H., Payne T., Berry J. O., Walsh J. A. & Carr J. P. A *Cucumber mosaic virus* mutant lacking the 2b counter-defence protein gene provides protection against wild-type strains. Journal of General Virology 88, 2862–2871 (2007). 17872541 10.1099/vir.0.83138-0

[b15] Ziebell H. & Carr J. P. Effects of dicer-like endoribonucleases 2 and 4 on infection of Arabidopsis thaliana by cucumber mosaic virus and a mutant virus lacking the 2b counter-defence protein gene. Journal of General Virology 90, 2288–2292 (2009). 19474248 10.1099/vir.0.012070-0

[b16] Ding S.-W. & Voinnet O. Antiviral immunity directed by small RNAs. Cell 130, 413–426 (2007). 17693253 10.1016/j.cell.2007.07.039 PMC2703654

[b17] Calarco J. P. & Martienssen R. A. Genome reprogramming and small interfering RNA in the *Arabidopsis* germline. Current Opinion in Genetics & Development 21, 134–139 (2011). 21330131 10.1016/j.gde.2011.01.014 PMC3073301

[b18] Chen X. Small RNAs—secrets and surprises of the genome. Plant Journal 61, 941–958 (2010). 20409269 10.1111/j.1365-313X.2009.04089.x PMC3062250

[b19] Folimonova S. Y. Developing an understanding of cross-protection by *Citrus tristeza virus*. Frontiers in Microbiology 4, 76 (2013). 23577008 10.3389/fmicb.2013.00076 PMC3616238

[b20] Folimonova S. Y. *et al.* Infection with strains of *Citrus Tristeza Virus* does not exclude superinfection by other strains of the virus. Journal of Virology 84, 1314–1325 (2010). 19923189 10.1128/JVI.02075-09 PMC2812332

[b21] Tscherne D. M. *et al.* Superinfection exclusion in cells infected with *Hepatitis C virus*. Journal of Virology 81, 3693–3703 (2007). 17287280 10.1128/JVI.01748-06 PMC1866098

[b22] Zou G. *et al.* Exclusion of *West Nile virus* superinfection through RNA replication. Journal of Virology 83, 11765–11776 (2009). 19726510 10.1128/JVI.01205-09 PMC2772679

[b23] Kobiler O., Lipman Y., Therkelsen K., Daubechies I. & Enquist L. W. Herpesviruses carrying a Brainbow cassette reveal replication and expression of limited numbers of incoming genomes. Nature Communications 1, 146 (2010). 10.1038/ncomms1145 PMC3079281 21266996

[b24] Qu F., Ye X. & Morris T. J. Arabidopsis DRB4, AGO1, AGO7, and RDR6 participate in a DCL4-initiated antiviral RNA silencing pathway negatively regulated by DCL1. Proceedings of the National Academy of Sciences USA 105, 14732–14737 (2008). 10.1073/pnas.0805760105 PMC2567185 18799732

[b25] Qu F., Ren T. & Morris T. J. The coat protein of Turnip crinkle virus suppresses posttranscriptional gene silencing at an early initiation step. Journal of Virology 77, 511–522 (2003). 12477856 10.1128/JVI.77.1.511-522.2003 PMC140649

[b26] Deleris A. *et al.* Hierarchical action and inhibition of plant Dicer-Like proteins in antiviral defense. Science 313, 68–71 (2006). 16741077 10.1126/science.1128214

[b27] Cao M. *et al.* The capsid protein of *Turnip crinkle virus* overcomes two separate defense barriers to facilitate systemic movement of the virus in *Arabidopsis*. Journal of Virology 84, 7793–7802 (2010). 20504923 10.1128/JVI.02643-09 PMC2897622

[b28] Zhang X., Zhang X., Singh J., Li D. & Qu F. Temperature-dependent survival of *Turnip crinkle virus*-infected *Arabidopsis* plants relies on an RNA silencing-based defense that requires DCL2, AGO2, and HEN1. Journal of Virology 86, 6847–6854 (2012). 22496240 10.1128/JVI.00497-12 PMC3393596

[b29] Carrington J. C., Kasschau K. D., Mahajan S. K. & Schaad M. C. Cell-to-cell and long distance transport of viruses in plants. Plant Cell 8, 1669–1681 (1996). 12239357 10.1105/tpc.8.10.1669 PMC161306

[b30] Harvey J. J. W. *et al.* An antiviral defense role of AGO2 in plants. PLoS ONE 6, e14639. 10.1371/journal.pone.0014639 (2011). 21305057 PMC3031535

[b31] Chiu W.-l. *et al.* Engineered GFP as a vital reporter in plants. Current Biology 6, 325–330 (1996). 8805250 10.1016/s0960-9822(02)00483-9

[b32] Haseloff J., Siemering K. R., Prasher D. C. & Hodge S. Removal of a cryptic intron and subcellular localization of green fluorescent protein are required to mark transgenic Arabidopsis plants brightly. Proceedings of the National Academy of Sciences USA 94, 2122–2127 (1997). 10.1073/pnas.94.6.2122 PMC20051 9122158

[b33] Monsion B., Froissart R., Michalakis Y. & Blanc S. Large bottleneck size in *Cauliflower mosaic virus* populations during host plant colonization. PLoS Pathogens 4, e1000174 (2008). 18846207 10.1371/journal.ppat.1000174 PMC2553192

[b34] Roberts A. G. *et al.* Phloem unloading in sink leaves of *Nicotiana benthamiana*: comparison of a fluorescent solute with a fluorescent virus. Plant Cell 9, 1381–1396 (1997). 12237387 10.1105/tpc.9.8.1381 PMC157005

[b35] Dietrich C. & Maiss E. Fluorescent labelling reveals spatial separation of potyvirus populations in mixed infected *Nicotiana benthamiana* plants. Journal of General Virology 84, 2871–2876 (2003). 13679622 10.1099/vir.0.19245-0

[b36] Takahashi T. *et al.* Analysis of the spatial distribution of identical and two distinct virus populations differently labeled with cyan and yellow fluorescent proteins in coinfected plants. Phytopathology 97, 1200–1206 (2007). 18943677 10.1094/PHYTO-97-10-1200

[b37] Zwart M. P., Daros J.-A. & Elena S. F. Effects of potyvirus effective population size in inoculated leaves on viral accumulation and the onset of symptoms. Journal of Virology 86, 9737–9747 (2012). 22740417 10.1128/JVI.00909-12 PMC3446627

[b38] Lin J. *et al.* The *Bean pod mottle virus* RNA2-encoded 58-Kilodalton protein P58 is required in cis for RNA2 accumulation. Journal of Virology 88, 3213–3222 (2014). 24390330 10.1128/JVI.03301-13 PMC3957913

